# Draft Genome Sequence of *Xylella fastidiosa* subsp. *fastidiosa* Strain Stag’s Leap

**DOI:** 10.1128/genomeA.00240-16

**Published:** 2016-04-21

**Authors:** J. Chen, F. Wu, Z. Zheng, X. Deng, L. P. Burbank, D. C. Stenger

**Affiliations:** a U.S. Department of Agriculture, Agricultural Research Service, San Joaquin Valley Agricultural Sciences Center, Parlier, California, USA; b College of Agriculture, South China Agricultural University, Guangzhou, Guangdong, People’s Republic of China

## Abstract

*Xylella fastidiosa* subsp. *fastidiosa* causes Pierce’s disease of grapevine. Presented here is the draft genome sequence of the Stag’s Leap strain, previously used in pathogenicity/virulence assays to evaluate grapevine germplasm bearing Pierce’s disease resistance and a phenotypic assessment of knockout mutants to determine gene function.

## GENOME ANNOUNCEMENT

*Xylella fastidiosa* is a Gram-negative xylem-limited bacterium (1, 2) comprising multiple subspecies causing disease in a wide variety of horticultural and landscape perennials (3). Pierce’s disease (PD) of grapevine is caused by strains of *X. fastidiosa* subsp. *fastidiosa*. Over the past 15 years, considerable effort has been devoted toward understanding mechanisms of *X. fastidiosa* subsp. *fastidiosa* pathogenicity and interactions with the grapevine host (4). Temecula1 (accession no. NC_004556.1) was the first strain of the subspecies to be completely sequenced (5); this sequence serves as a reference for PD research. The complete genome of *X. fastidiosa* subsp. *fastidiosa* strain M23 (accession no. NC_010577.1), isolated from almonds in San Joaquin Valley, CA, also was sequenced (6) and is highly similar to that of Temecula1. A third strain of *X. fastidiosa* subsp. *fastidiosa*, Stag’s Leap (isolated from grapevine in Napa Valley, CA) (7), has been used to evaluate PD resistant germplasm and in knockout/pathogenicity/virulence assays to elucidate gene function (8). This study fills a critical knowledge gap, the draft genome sequence of strain Stag’s Leap.

For sequencing, Stag’s Leap was cultured in periwinkle wilt medium (9) at 28°C for 14 days. Cells were collected; total genomic DNA was extracted according to a standard procedure (10). Whole-genome sequencing was performed on the Illumina MiSeq platform (Illumina, Inc., San Diego, CA); 6.59 × 10^6^ paired-end reads (301 bp average length) were generated. Sequence reads were assembled *de novo* with CLC Genomics Workbench (version 7.5), yielding 99 contigs >1,000 bp. The same sequence reads were then mapped to the M23 complete genome using Bowtie 2 version 2.2.6 (11), generating 20 contigs. Finally, contigs from both *de novo* and mapping methods were combined manually. The final assembled draft genome (750× mean coverage) had a G+C content of 51.7%, with 2,510,798 bp distributed among 15 contigs ranging in size from 1,307 bp to 731,756 bp. All sequences were annotated using the RAST server (http://rast.nmpdr.org/) (12). The chromosomal sequence had 2,756 open reading frames (ORFs) and 55 RNA genes. The Stag’s Leap draft chromosomal sequence corresponds to 99.0% of the M23 genome (2,535,690 bp) and 99.6% of the Temecula1 genome (2,519,802 bp).

### Nucleotide sequence accession numbers.

The *X. fastidiosa* subsp. *fastidiosa* strain Stag’s Leap whole-genome shotgun project has been deposited at DDBJ/EMBL/GenBank under accession no. LSMJ00000000. The version described in this paper is version LSMJ00000000.1.

## References

[1] Wells JM, Raju BC, Hung H-Y, Weisburg WG, Mandelco-Paul L, Brenner DJ 1987 *Xylella fastidiosa* gen. nov., sp. nov.: Gram-negative, xylem-limited, fastidious plant bacteria related to *Xanthomonas* spp. Int J Syst Bacteriol 37:136–143. doi:10.1099/00207713-37-2-136.

[2] Schaad NW, Postnikova E, Lacy G, Fatmi M, Chang CJ 2004 *Xylella fastidiosa* subspecies: *X. fastidiosa* subsp. [correction] *fastidiosa* [correction] subsp. nov., *X. fastidiosa* subsp. *multiplex* subsp. nov., and *X. fastidiosa* subsp. *pauca* subsp. nov. Syst Appl Microbiol 27:290–300. doi:10.1078/0723-2020-00263. 15214634

[3] Almeida RPP, Nunney L 2015 How do plant diseases caused by *Xylella fastidiosa* emerge? Plant Dis 99:1457–1467. doi:10.1094/PDIS-02-15-0159-FE. 30695952

[4] Retchless A, Labroussaa F, Shapiro L, Stenger DC, Lindow SE, Almeida RPP 2014 Genomic insights into *Xylella fastidiosa* interactions with plant and insect hosts, p 177–202. In Gross DC, Lichens-Park A, Kole C (ed), Genomics of plant-associated bacteria. Springer-Verlag, Berlin, Germany.

[5] Van Sluys MA, de Oliveira MC, Monteiro-Vitorello CB, Miyaki CY, Furlan LR, Camargo LEA, da Silva ACR, Moon DH, Takita MA, Lemos EGM, Machado MA, Ferro MIT, da Silva FR, Goldman MHS, Goldman GH, Lemos MVF, El-Dorry H, Tsai SM, Carrer H, Carraro DM, de Oliveira RC, Nunes LR, Siqueira WJ, Coutinho LL, Kimura ET, Ferro ES, Harakava R, Kuramae EE, Marino CL, Giglioti E, Abreu IL, Alves LMC, do Amaral AM, Baia GS, Blanco SR, Brito MS, Cannavan FS, Celestino AV, da Cunha AF, Fenille RC, Ferro JA, Formighieri EF, Kishi LT, Leoni SG, Oliveira AR, Rosa VE Jr, Sassaki FT, Sena JAD, de Souza AA, Truffi D, et al. 2003 Comparative analyses of the complete genome sequences of Pierce’s disease and citrus variegated chlorosis strains of *Xylella fastidiosa*. J Bacteriol 185:1018–1026. doi:10.1128/JB.185.3.1018-1026.2003. 12533478 PMC142809

[6] Chen J, Xie G, Han S, Chertkov O, Sims D, Civerolo EL 2010 Whole genome sequences of two *Xylella fastidiosa* strains (M12 and M23) causing almond leaf scorch disease in California. J Bacteriol 192:4534. doi:10.1128/JB.00651-10. 20601474 PMC2937377

[7] Buzkana N, Kocsis L, Walker MA 2005 Detection of *Xylella fastidiosa* from resistant and susceptible grapevine by tissue sectioning and membrane entrapment immunofluorescence. Microbiol Res 160:225–231. doi:10.1016/j.micres.2004.05.006. 16035233

[8] Burbank LP, Stenger DC 2016 A temperature-independent cold-shock protein homolog acts as a virulence factor in *Xylella fastidiosa*. Mol Plant Microbe Interact, in press. doi:10.1094/MPMI-11-15-0260-R. 26808446

[9] Davis MJ, French WJ, Schaad NW 1981 Axenic culture of the bacteria associated with phony disease of peach and plum leaf scald. Curr Microbiol 6:309–314. doi:10.1007/BF01566883.

[10] Maniatis T, Fritsch EF, Sambrook J 1982 Molecular cloning: a laboratory manual. Cold Spring Harbor Laboratory Press, Cold Spring Harbor, NY.

[11] Langmead B, Salzberg SL 2012 Fast gapped-read alignment with Bowtie 2. Nat Methods 9:357–359. doi:10.1038/nmeth.1923. 22388286 PMC3322381

[12] Aziz RK, Bartels D, Best AA, DeJongh M, Disz T, Edwards RA, Formsma K, Gerdes S, Glass EM, Kubal M, Meyer F, Olsen GJ, Olson R, Osterman AL, Overbeek RA, McNeil LK, Paarmann D, Paczin T, Parrello B, Pusch GD, Reich C, Stevens R, Vassieva O, Vonstein V, Wilke A, Zagnitko O 2008 The RAST server: Rapid Annotations using Subsystems Technology. BMC Genomics 9:75. doi:10.1186/1471-2164-9-75. 18261238 PMC2265698

